# Supplementary material for: DMRforPairs: identifying Differentially Methylated Regions between unique samples using array based methylation profiles
Source: BMC Bioinformatics. 2014 May 15;15:141. doi: 10.1186/1471-2105-15-141 (PMC4046028; doi:10.1186/1471-2105-15-141)
Supplement: Additional file 2 — DMRforPairs output for the comparison of A431-MCF7 and NA17018-NA17105. Please start from the HTML files in each folder. Available via the BMC Bioinformatics website. [file 1471-2105-15-141-S2.zip › 1394847754114233_MOESM2_ESM/A431_MCF7/figures/39827.pdf]

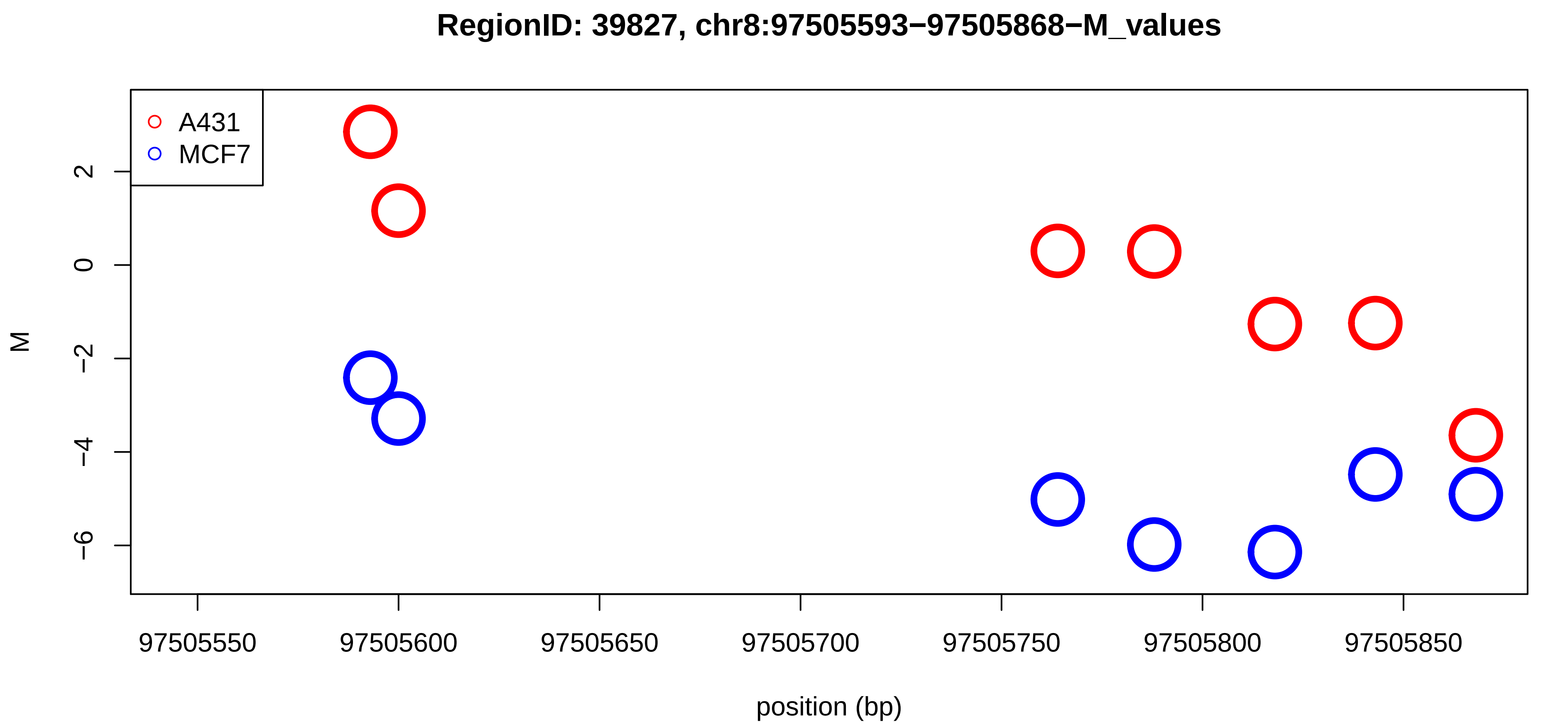

RegionID: 39827, chr8:97505593−97505868−M_values
A431
MCF7
M
2
0
−2
−4
−6
97505550
97505600
97505650
97505700
97505750
97505800
97505850
position (bp)

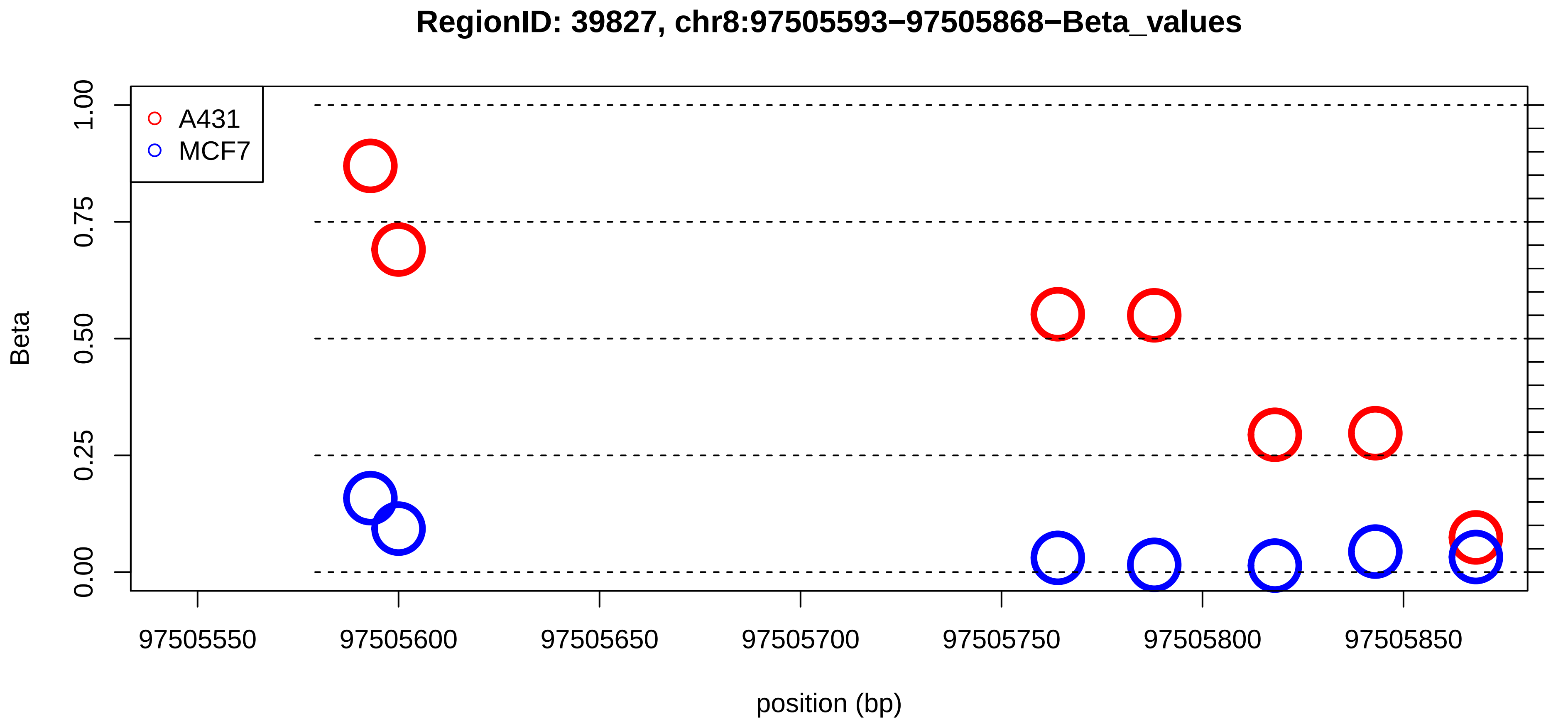

RegionID: 39827, chr8:97505593−97505868−Beta_values
A431
MCF7
Beta
1.00
0.75
0.50
0.25
0.00
97505550
97505600
97505650
97505700
97505750
97505800
97505850
position (bp)

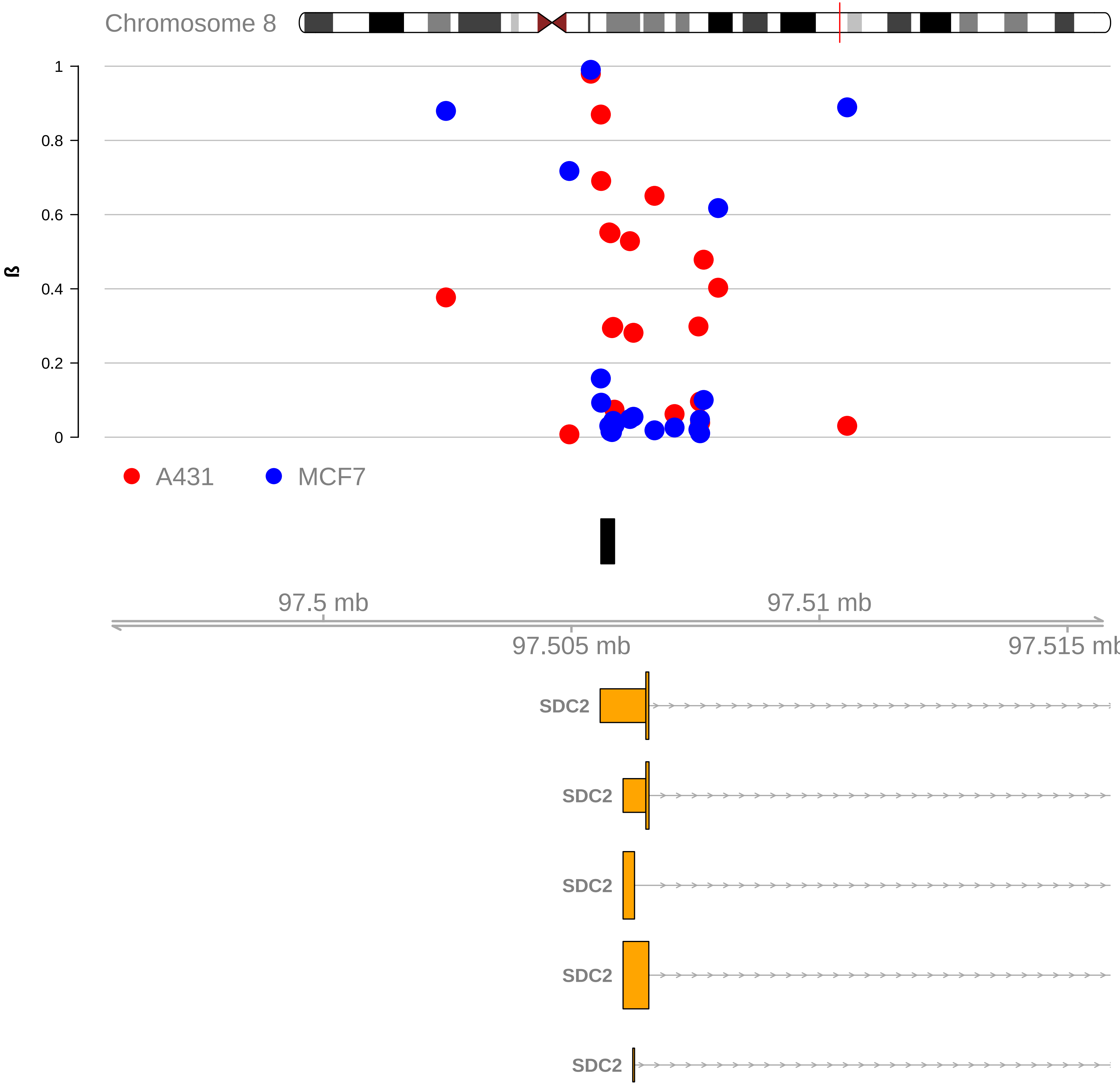

Chromosome 8
1
0.8
0.6
0.4
0.2
0
ß
A431
MCF7
97.5 mb
97.51 mb
97.505 mb
97.515 mb
SDC2
SDC2
SDC2
SDC2
SDC2
